# Degenerative findings in lumbar spine MRI: an inter-rater reliability study involving three raters

**DOI:** 10.1186/s12998-020-0297-0

**Published:** 2020-02-11

**Authors:** Klaus Doktor, Tue Secher Jensen, Henrik Wulff Christensen, Ulrich Fredberg, Morten Kindt, Eleanor Boyle, Jan Hartvigsen

**Affiliations:** 1 Department of Sport Sciences and Clinical Biomechanics, University of Southern Denmark, Odense, Denmark; 2 Nordic Institute of Chiropractic and Clinical Biomechanics, Odense, Denmark; 3 Diagnostic Centre, University Research Clinic for Innovative Patient Pathways, Silkeborg Regional Hospital, Aarhus University, Odense, Denmark; 4 Department of Clinical Medicine, Aarhus University, Odense, Denmark; 5 The Rheumatology Research Unit, Odense University Hospital, University of Southern Denmark, Odense, Denmark

**Keywords:** Agreement, Reliability, Reproducibility, Lumbar spine, Low Back pain, Leg pain, Sciatica, No-low back pain, Recumbent MRI, Supine MRI, MR, Magnetic resonance imaging

## Abstract

**Background:**

For diagnostic procedures to be clinically useful, they must be reliable. The interpretation of lumbar spine MRI scans is subject to variability and there is a lack of studies where reliability of multiple degenerative pathologies are rated simultaneously. The objective of our study was to determine the inter-rater reliability of three independent raters evaluating degenerative pathologies seen with lumbar spine MRI.

**Methods:**

Fifty-nine people, 35 patients with low back pain (LBP) or LBP and leg pain and 24 people without LBP or leg pain, received an MRI of the lumbar spine. Three raters (one radiologist and two chiropractors) evaluated the MRIs for the presence and severity of eight degenerative spinal pathologies using a standardized format: Spondylolisthesis, scoliosis, annular fissure, disc degeneration, disc contour, nerve root compromise, spinal stenosis and facet joint degeneration. Findings were identified and classified at disc level according to type and severity. Raters were instructed to evaluate all study sample persons once to assess inter-rater reliability (fully crossed design). Reliability was calculated using Gwet’s Agreement Coefficients (AC_1_ and AC_2_) and Cohen’s Kappa (κ) and Conger’s extension of Cohen’s. Gwet’s probabilistic benchmarking method to the Landis and Koch scale was used. MRI-findings achieving substantial reliability was considered acceptable.

**Results:**

Inter-rater reliability for all raters combined, ranged from (Gwet’s AC_1_ or AC_2_): 0.64–0.99 and according to probabilistic benchmarking to the Landis and Koch scale equivalent to moderate to almost perfect reliability. Overall reliability level for individual pathologies was almost perfect reliability for spondylolisthesis, spinal stenosis, scoliosis and annular fissure, substantial for nerve root compromise and disc degeneration, and moderate for facet joint degeneration and disc contour.

**Conclusion:**

Inter-rater reliability for 3 raters, evaluating 177 disc levels, was found to be overall acceptable for 6 out of 8 degenerative MRI-findings in the lumbar spine. Ratings of facet joint degeneration and disc contour achieved moderate reliability and was considered unacceptable.

## PACS picture, archiving and communication system (i.e. Agfa Impax)

REDCap Research Electronic Data Capture program

## Background

Imaging has been used in the diagnostic workup of people seeking care for low back pain for more than a hundred years. Consequently, conventional radiographs, computerized tomography (CT) or magnetic resonance imaging (MRI) is accepted, if not expected, by many patients and doctors [1–4]. For any diagnostic procedure including imaging to be useful, it must first demonstrate adequate reliability [5, 6].

Most people would claim that they understand and appreciate the meaning of agreement as well as the meaning of disagreement, and we all deal with the consequences of both every single day of our lives. In healthcare this is certainly the case and can have grave consequences for doctors and patients, since the quality of care and procedures delivered in health care systems around the world, depends on this seemingly simple notion. Patients are increasingly aware of different doctors having different opinions regarding their health issues. This variability may be due to variations in nomenclature [7, 8], and it is critical for radiologists and other interpreters of diagnostic imaging, such as chiropractors, to reduce interpreter variability [6]. Inter-rater reliability is an important parameter to measure and is of concern as data-sets should reflect variation in the study participants and not variation in the raters involved in the study. For valid results it is important that raters are updated and trained in the use of standardized protocols prior to the evaluation of MRI findings. Diagnostic classification systems for MRI have been proposed to address interpreter variability and in a systematic review the reliability of different nomenclature and grading systems for lumbar disc herniation and nerve root compression were compared, ranging from κ = 0.39–0.81 [9], and representing quite a range in reliability despite limited to two degenerative conditions: Disc herniation and nerve root compromise. It is uncertain what kind of variability and reliability raters would produce if evaluating multiple degenerative pathologies simultaneously. Previous reliability studies of MRI findings of the lumbar spine have mainly reported on single findings or a specific grading scale of lumbar spine degenerative pathology, such as disc herniation [10, 11], spinal stenosis [7, 12] and end-plate changes [13]. However, a few studies have examined a handful of spinal degenerative pathologies simultaneously using various classification systems/scales [5, 8, 14, 15]. To our knowledge, there are few reliability studies on lumbar spine MRI findings that have both included raters of different professions and included multiple degenerative findings: One study compared medical radiologists, chiropractic radiologists and chiropractors [16]. Another study investigated reliability between a radiologist, a chiropractor and a second year resident of rheumatology in classifying degenerative MRI-findings of the cervical spine [17]. Thus, there is a need for studies investigating reliability for a wider range of spinal degenerative pathologies based on standardized formats and involving more than one profession participating in MRI readings in radiology departments.

### Objectives

The objective of this study was to determine the inter-rater reliability of the evaluation of degenerative findings in lumbar spine MRI.

## Methods

### Design

Fully crossed inter-rater reliability study.

Guidelines for reporting reliability and agreement studies (GRRAS-guidelines) have been followed in this paper [18].

### Sample size calculation

In a test for agreement between two raters using the Kappa statistic, a sample size of 51 subjects achieves 80% power to detect a true Kappa value of 0.70 in a test of H0: Kappa = κ0 vs. H1: Kappa ≠ κ0, when there are 6 categories with frequencies equal to 0.48, 0.28, 0.20, 0.03, 0.01, and 0.00. This power calculation is based on a significance level of 0.05000. Furthermore, we assumed the three disc-levels per participant to be independent, leaving us with 177 observations [19].

### Study population and reliability sample

Fifty-nine MRIs of the lumbar spine from people with or without LBP, who were enrolled in a cross-sectional study, were included in this study from February 26th, 2018 to April 26th, 2018.

People with LBP or LBP and leg pain were invited to participate, when scheduled to the hospital for an MRI procedure, ordered by their primary care physician. The inclusion/exclusion criteria for people with LBP were: 1) Referred to Department of Radiology, Silkeborg Regional Hospital, Denmark for MRI from primary care (general practitioners or chiropractors) with LBP or LBP and leg pain; 2) Having symptoms for > 4 weeks; 3) 18–60 years of age; 4) Not part of a referral pathway to spinal surgery or another secondary care sector activity 5) No suspicion of serious pathology, i.e. cancer, infection or inflammatory arthritis; 6) Able to stand up for at least 20 min; 7) Able to read and write Danish.

The same criteria were used for people without LBP (no-LBP) with the exclusion of criteria 1) and 2). People in the no-LBP group were mainly recruited from a local school of nursing near the hospital, employees at the hospital and through announcements in workplace environments in Silkeborg Municipality/City, Denmark. Recruitment was carried out by posters and by personal communication.

**Fig. 1:**
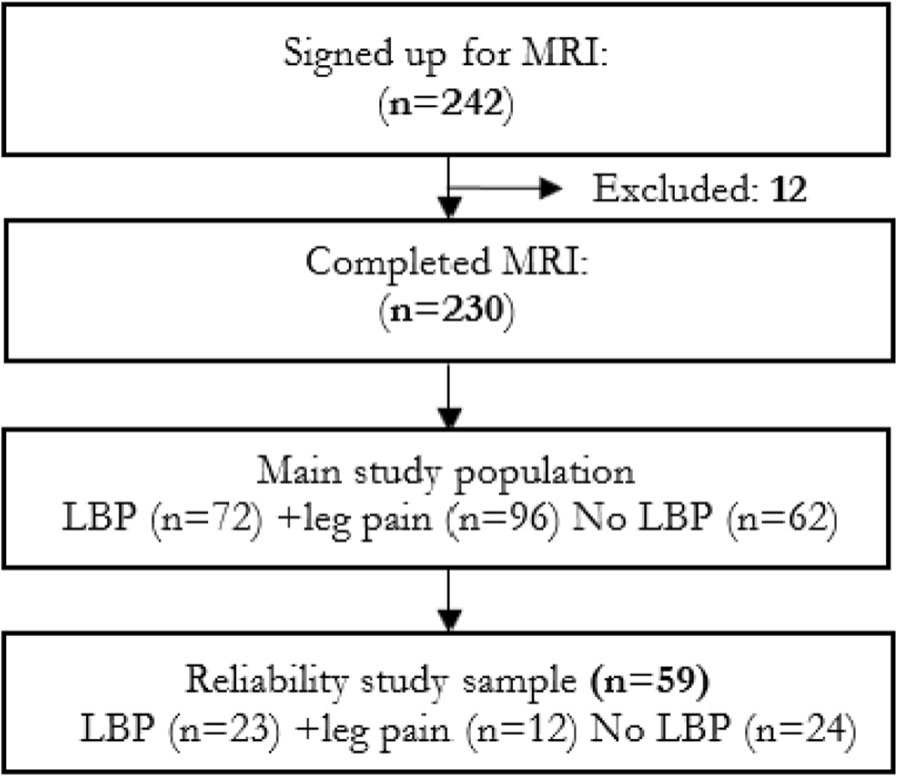
Flowchart of selection of main study population and the reliability study sample

Informed consent was provided by all participants via REDCap (Research Electronic Data Capture) installed on i-Pads. Recruitment for all participants was consecutive on a first come, first serve basis. A total of 242 people were initially included in the study population. Participants were assigned to subgroups based on self-reported symptoms/no-symptoms in their baseline questionnaires. Six people were excluded because of age over 60 years and 6 were excluded because of technical problems with their baseline questionnaires or failing to complete the MRI procedures. The remaining 230 individuals defined our main study population (see Fig. 1), and the first 59 individuals giving a fair representation of participants with LBP, leg pain and no-LBP in each group defined the reliability study sample.

### MRI-procedures

All patients were scanned in either a Siemens Avanto 1.5 T or a Siemens Skyra 3 T MRI unit and all no-LBP individuals were scanned in an open MRI unit (Paramed OpenMR 0.5 T). MRI sequences for all three scanners were: Sagittal T1 and T2 as well as T2 axial at the three lowest lumbar levels. For the 1.5 T and 3 T systems, the sagittal T2 weighted sequence also included T2 fatsat (DIXON).

### Raters, training and consensus

Rater 1, a medical radiologist consultant with 30 years of experience in musculoskeletal MRI; Rater 2, a chiropractor and PhD student with 28 years of clinical and radiography experience and 4 years of MRI experience including over 1000 supervised reports of lumbar MRI in the same radiology department; Rater 3, a chiropractor and senior researcher with 12 years of clinical research and MRI experience from radiology departments. All raters had, in various degrees, experience with reliability of diagnostic classification model as well as clinical experience with spinal diagnostic imaging [13, 17, 20, 21].

To ensure consensus regarding understanding of the diagnostic classification, an evaluation manual was prepared based on existing literature [5, 22–29] (see Additional files 1 and 2). For the purpose of training and to identify practical issues in the evaluation process, all three raters independently analyzed and classified 10 MRIs based on the manual. The raters then met to compare ratings and discuss adjustments to the assessment and coding process. The evaluation manual was then adjusted, and a second set of 5 MRIs was rated, compared and discussed before the final version of the manual was prepared.

### MRI evaluation and classification of findings

The three raters evaluated 177 disc levels (the three lowest lumbar levels: L3/L4 – L5/S1) for the presence of the following eight degenerative pathologies, independently: Spondylolisthesis; Scoliosis; Annular fissure; Disc degeneration; Disc contour; Nerve root compromise; Spinal stenosis; and Facet joint degeneration. The image findings were classified based on methods described in the literature (see Table 1).

**Table 1:** Classification of MRI findings

Diagnostic findings	Scale/categories	Definitions
Spondylolisthesis, (Meyerding [27])	Ordinal	Defined as slippage of the vertebral body in relation to the one below in: Anterior, posterior or lateral direction.
	0	Normal
Grade I	1	Displacement of vertebral body ≤ ¼ of vertebral body below.
Grade II	2	Displacement of vertebral body ≤ ½ of vertebral body below.
Grade III	3	Displacement of vertebral body ≤ ¾ of vertebral body below.
Grade IV	4	Displacement of vertebral body ≤ 4/4 of vertebral body below.
Disc degeneration, (Pfirrmann [22])	Ordinal	For this study grade I and II is considered normal.
Grade I:	0	Nucleus pulposus is homogenous and has high, bright white, signal intensity. Clear distinction of nucleus and annulus. Normal heights of the intervertebral disk
Grade II:	0	Like grade I, but the nucleus pulposus is inhomogeneous, with or without clear horizontal bands.
Grade III:	1	Nucleus pulposus being inhomogeneous and gray, unclear distinction of the nucleus and annulus, intermediate signal intensity and normal to slightly decreased intervertebral disc height.
Grade IV:	2	Inhomogeneous, gray to black nucleus pulposus and no distinction between the nucleus and the annulus. The signal intensity is intermediate to hypointense and normal to moderately decreased disc height.
Grade V:	3	Nucleus pulposus is inhomogeneous and black, with hypointense signal intensity and collapsed disk space.
Nerve root compromise, (Lee [24])	Ordinal	
Normal	0	No contact to nerve roots
Contact	1	Perineural fat obliteration from two opposing sides. No morphologic change (no signs of compression/deformation) of the nerve root.
Contact and deviation	2	Perineural fat obliteration surrounding the nerve root from four sides. No morphologic change (no compression/deformation) of nerve root.
Compression	3	Visible nerve root collapse or morphologic change
Spinal stenosis, (Lee [24])	Ordinal	
Central		
No stenosis:	0	Up to 3 mm disc bulge is normal.
Relative stenosis:	1	Reduced space < 50%, but still visible fluid signal around the nerve roots.
Absolute stenosis:	2	50% reduction or more of the dural sac area and no visible signal (dark/black) from cerebrospinal fluid around the nerve roots or medulla spinalis.
Lateral		
No stenosis:	0	Normal levels of perineural fat.
Relative stenosis:	1	Reduced space, perineural fat obliteration from at least two opposing sides but still visible perineural fat/CSF signal in the recess.
Absolute stenosis:	2	Reduction of the recess to a point where perineural fat signal/CSF signal no longer is visible.
Foraminal		
No stenosis:	0	Normal upside-down pear shape contour of the foramina with an apical nerve root location.
Relative stenosis:	1	Reduced space, but still visible perineural fat signal in the foramen.
Absolute stenosis:	2	Reduction of the foramen to the point where perineural fat signal is no longer visible.
Facet degeneration,		
(Ross/Moore [30]; Pathria [31])	Ordinal	
No degeneration:	0	Normal
Mild degeneration:	1	Mild joint space narrowing and joint irregularity.
Moderate degeneration:	2	Moderate joint space narrowing/irregularity, subchondral sclerosis/osteophyte formation.
Severe degeneration:	3	Little, if any, joint space, severe subchondral sclerosis/ osteophyte formation. Possible subluxation and/or subchondral cyst formation.
Scoliosis (Cobb [29])	Binominal	Defined as any spinal curvature with a Cobb’s angle greater than 10 degrees.
sinistro convex	0/1	Apex of the curvature to the left.
dextro convex	0/1	Apex of the curvature to the right.
rotational	0/1	Pedicles and spinous process oriented to the left or right.
Annular Fissure, (April [23])	Binominal 0/1	High T2 signal (HIZ) in the otherwise low signal annulus. Diameter > 1.5 mm. Annulus material visible all around the fissure.
Disc contour, (Fardon [32])	Nominal	
Normal or bulge	0	< 3 mm and > 25% of the disc periphery (90 degrees). Negative for herniation.
Protrusion:	1	< 25% (90 degrees) of disc periphery, distance between disco-vertebral corners is greater than distance of disc material past the base, measured in same plane.
Extrusion:	2	Dimension of disc material in any one direction is greater than distance between disco-vertebral corners. Migration cephalad or caudad indicates extrusion.
Sequestration:	3	Disc material has lost continuity with the parent disc.
Combination of types	4	Combined protrusion and extrusion

The raters retrieved images in PACS (Picture, Archiving and Communication System: Agfa Impax, version 5.2) and filled in the standardized research evaluation form in REDCap. All images were assessed and analyzed on diagnostic Agfa Impax workstations with high resolution diagnostic monitors (Totoku Monochrome MS33I2_Pair, 3 mpx. Barco MDNC-2121 color pair, 2 mpx and Barco MDNC-2121 monochrome pair, 2 mpx). The raters were blinded with respect to clinical information and previous report of findings.

### Data management and statistical analysis

Data analysis was carried out in Stata, ver.15.1 (StataCorp LLC, 4905 Lakeway Drive, College Station, Texas 77,845, USA) and AgreeStat 2015.1 for Excel Windows/Mac (Advanced Analytics, LLC. PO Box 2696, Gaithersburg, MD 20886–2696, USA.).

Inter-rater reliability, based on 3 lower disc level of 59 persons = 177 levels, was determined for binominal, nominal and ordinal data (see Table 1) by calculating percent agreement and chance-corrected agreement coefficients (Cohen’s/Conger’s κ and Gwet’s AC_1_ (unweighted) and AC_2_ (weighted)) for pair-wise raters and for the three raters overall [33], and were reported with 95% confidence intervals. Gwet’s agreement coefficients, AC_1_ and AC_2_, were used to address the κ paradox [34] and has been shown to be more stable and paradox-resistant than Cohen’s κ and other coefficients [33, 35]. The κ paradoxes with very low or very high prevalence’s are well described in the literature [36, 37]. The first paradox occurs when percent chance agreement (p_e_), is large, the correction process can convert a relatively high value of observed agreement (p_O_) into a relatively low value of κ. The second paradox occurs when unbalanced marginal totals produce higher values of κ than more balanced totals. In order to deal with the paradoxes (very low or very high prevalence’s), we used Gwet’s Agreement Coefficients (AC_1_ and AC_2_). In order to compare our results with previous literature, we chose to also present both Cohen/Conger’s κ as well as the benchmarking procedure to the Landis and Koch scale [38]. The probabilistic method for benchmarking, as suggested by Gwet, is the absolute agreement and chance corrected agreement coefficients benchmarked as the cumulative probability (in our case exceeding 95%) for the any coefficient to fall into one of the following intervals: < 0.00 “Poor”; 0.01 to 0.20 “Slight”; 0.21 to 0.40 “Fair”; 0.41 to 0.60 “Moderate”; 0.61 to 0.80 “Substantial” and 0.81 to 1.00 “Almost Perfect” [39]. This method allows for a direct and more precise comparison of different agreement coefficients and their representation on the Landis and Koch scale (or any other scale used). Substantial reliability (0.61–0.80) was defined as acceptable for the purpose of this study and confidence intervals were presented with 95% certainty to include the true estimate. We recommend interested readers to follow the link in the reference list for more insight and comprehension of Gwet’s Agreement Coefficents compared to other coefficients and the probabilistic benchmarking [40]. Key characteristics for the study target population and the study sample are presented for age, gender, symptoms, duration (see Table 2).

**Table 2:** Characteristics of the study target population and study sample for reliability

Characteristics	Cross-sectional study population (*N* = 230)	Reliability study sample Inter-rater analysis (*n* = 59)
Age, in years, mean	42.1 (SD 12.1)	38.1 (SD 14.1)
Females, n(%)	118 (51.1%)	27 (45.8%)
Patients, LBP, n(%)	72 (31.3%)	23 (39.0%)
Patients, LBP + leg pain, n(%)	96 (41.7%)	12 (20.3%)
Symptoms > 4 wks., n(%)	168 (73.0%)	35 (59.3%)
No LBP persons, n(%)	62 (27.0%)	24 (40.7%)

## Results

The mean age is 42 years for the target population and 38 years for the reliability sample. Women represents 51 and 46% of the study target population and reliability sample respectably. LBP and leg pain patients account for 73 and 59% respectably, all with symptoms over 4 weeks. No-LBP persons accounted for 27 and 41% respectively.

The prevalence of positive findings for all raters can be found in contingency tables in additional files. Generally, rater 1 had lower prevalence of diagnostic findings (average = 0.26), compared to rater 2 (average = 0.40) and rater 3 (average = 0.44), with a total prevalence ranging from: 0.05–0.80 for the individual MR-findings.

In Table 3, results for reliability at disc levels are presented. The overall reliability (raters 1,2 and 3) for the eight spinal degenerative pathologies ranged from moderate to almost perfect. Overall reliability level for individual pathologies was almost perfect for spondylolisthesis, spinal stenosis, scoliosis and annular fissure; substantial for nerve root compromise and disc degeneration; and moderate for facet joint degeneration and disc contour (prevalence of MRI-findings at disc levels can be found in Additional files 1 and 2 for ordinal and nominal scales respectively).

**Table 3:** Inter-rater reliability coefficients and percent agreement with probabilistic benchmarking to the Landis and Koch scale in classification of MRI-findings at disc level

Diagnostic finding *N* = 177 disc levels	Reliability Rater 1 vs. 2	Reliability Rater 1 vs. 3	Reliability Rater 2 vs. 3	All	Landis and Koch scale
	95% C.I.	95% C.I.	95% C.I.		Probabilistic benchmark
Spondylolisthesis					
Conger’s K	0.24 [−0.16:0.64]	0.36 [−0.01:0.72]	0.36 [− 0.01:0.72]	0.33	Slight
Gwet’s AC_2_	0.998 [0.997:1.000]	0.998 [0.996:0.999]	0.998 [0.996:0.999]	0.99	Almost perfect
%-agreement	0.998 [0.997:1.000]	0.998 [0.996:0.999]	0.998 [0.996:0.999]	0.99	Almost perfect
Disc degeneration					
Conger’s K	0.60 [0.51:0.70]	0.67 [0.58:0.76]	0.76 [0.69:0.82]	0.68	Moderate
Gwet’s AC_2_	0.90 [0.87:0.94]	0.89 [0.85:0.93]	0.91 [0.88:0.95]	0.90	Substantial
%-agreement	0.95 [0.93:0.96]	0.94 [0.93:0.96]	0.96 [0.95:0.97]	0.95	Substantial
Nerve compromise					
Conger’s K	0.55 [0.38:0.71]	0.56 [0.39:0.72]	0.52 [0.34:0.70]	0.54	Fair
Gwet’s AC_2_	0.96 [0.93:0.98]	0.93 [0.90:0.96]	0.92 [0.89:0.96]	0.93	Substantial
%-agreement	0.96 [0.95:0.98]	0.95 [0.93–0.97]	0.94 [0.92:0.97]	0.95	Substantial
Spinal stenosis					
Conger’s K	0.19 [0.08:0.29]	0.33 [0.22:0.45]	0.43 [0.34:0.53]	0.33	Fair
Gwet’s AC_2_	0.98 [0.97:0.98]	0.98 [0.98:0.99]	0.98 [0.97:0.98]	0.98	Almost perfect
%-agreement	0.98 [0.98:0.99]	0.99 [0.98:0.99]	0.98 [0.98:0.99]	0.98	Almost perfect
Facet degeneration					
Conger’s K	0.27 [0.16:0.38]	0.32 [0.21:0.42]	0.35 [0.25:0.46]	0.32	Slight
Gwet’s AC_2_	0.79 [0.74:0.84]	0.79 [0.74:0.84]	0.76 [0.71:0.82]	0.78	Moderate
%-agreement	0.88 [0.86:0.90]	0.89 [0.86–0.91]	0.87 [0.85:0.90]	0.88	Moderate
Scoliosis					
Cohen’s K	0.49 [0.06:0.92]	0.59 [0.22:0.96]	0.75 [0.40:1.00]	0.61	Fair
Gwet’s AC_1_	0.98 [0.96:1.00]	0.98 [0.96:1.00]	0.99 [0.97:1.00]	0.98	Almost perfect
%-agreement	0.98 [0.96:1.00]	0.98 [0.96:1.00]	0.99 [0.97:1.00]	0.98	Almost perfect
Annular Fissure					
Cohen’s K	0.50 [0.32:0.68]	0.45 [0.26:0.65]	0.61 [0.45:0.77]	0.53	Moderate
Gwet’s AC_1_	0.87 [0.82:0.93]	0.88 [0.82:0.93]	0.88 [0.83:0.93]	0.88	Almost perfect
%-agreement	0.88 [0.83:0.93]	0.88 [0.83:0.93]	0.89 [0.84:0.93]	0.88	Almost perfect
Disc contour					
Cohen’s K	0.36 [0.25:0.48]	0.27 [0.17:0.38]	0.39 [0.29:0.49]	0.34	Fair
Gwet’s AC_1_	0.73 [0.65:0.80]	0.59 [0.50:0.68]	0.62 [0.53:0.70]	0.64	Moderate
%-agreement	0.75 [0.69:0.82]	0.64 [0.57:0.71]	0.67 [0.60:0.74]	0.69	Substantial

Inter-rater reliability using Gwet’s AC_1_ (binominal/nominal data) and AC_2_ (ordinal data) and percent agreement are presented

For comparison Cohen’s K (binominal/nominal data) and Conger’s K (ordinal data) also presented

Numbers in parentheses are 95% confidence intervals [95% CI]

For the 3 rater pairs individually, the reliability ranged from moderate to almost perfect. For disc contour, there was a difference of one benchmark level between rater pairs, from moderate to substantial. There was no difference in benchmark levels between rater pairs for the remaining 7 pathologies.

The reliability among all 3 raters was moderate for facet joint degeneration and disc contour. Reliability for rater pairs 1–3 and 2–3 was also moderate for disc contour.

There was almost perfect reliability and very little variability between rater pairs for spondylolisthesis, spinal stenosis, scoliosis and annular fissure. There was substantial reliability for disc degeneration and nerve root compromise. Variability was highest between rater-pairs for disc contour, but low for the other MRI findings.

## Discussion

Inter-rater reliability for three rater-pairs was found overall acceptable for 6 of 8 degenerative MRI-findings of the lumbar spine. In addition, our results indicate that experienced chiropractors can achieve the same level of reliability as medical radiologists for MRI interpretations of spinal degenerative pathologies. Thus these classifications of findings are sufficiently comprehensible to be applied by experienced health care professionals and can be used for both quality assurance and research purposes.

Prior studies have investigated the reliability of identifying degenerative MRI-findings of the lumbar spine [38–41], but few are directly comparable to our study, because they investigated only one spinal degenerative pathology. Zoete et al. compared experienced medical and chiropractic radiologists reviewing MRI for lumbar spinal degenerative pathology [16]. The findings were dichotomized into a classification between “Specific findings” or “No specific findings”, and higher reliability was found with more experienced raters. Specialists regardless of professional background obtained the best results (moderate reliability). Moll et al. investigated the reliability between a radiologist, a chiropractor and a second year resident of rheumatology, in classifying degenerative MRI-findings of the *cervical* spine and found overall substantial interrater reliability (κ ≥ 0.61) [17]. In our study, we achieved very low variability between the 3 raters and only one of eight pathologies had a difference in reliability among raters of one benchmark level indicating that experienced chiropractors and medical radiologists can achieve acceptable reliability in MRI interpretations, even when evaluating for a range of spinal degenerative pathologies in the lumbar spine.

Carrino et al. is one of few studies that has examined inter-rater agreement across several different spinal pathologies (spondylolisthesis, disc degeneration, end-plate changes, annular fissure and facet degeneration) and including 111 cases [5]. Kappa values were generally modest and ranged for all raters overall from, κ: 0.43–0.66 (CI 0.27–0.70), with only disc degeneration being of acceptable reliability, κ > 0.60. Average overall kappa among raters was, κ = 0.53.

Another similar interrater agreement study involving 75 cases by Fu et al., included 10 degenerative spinal pathologies of the lumbar spine and reported absolute agreement and Fleiss κ, with κ-values being modest and with significant variability across degenerative conditions, ranging from 0.28–0.62 (CI 0.27–0.64) [8]. Excluding transitional vertebrae (κ = 0.62), all remaining 9 degenerative conditions in this study achieved unacceptable reliability, κ < 0.60. The overall average kappa coefficient among all 4 raters was κ = 0.43.

Absolute agreement and agreement coefficients in our study were similar or higher compared with Carrino et al., and generally higher compared with Fu et al. We achieved average overall κ = 0.59 among all 3 raters. Both studies had interdisciplinary representation of raters and provided training and evaluation manuals, but also based reliability measures on less robust kappa-statistics, so their result might have proved better than ours, if the method proposed by Gwet had been used.

### Methodical considerations

When a reliability study is based on samples smaller than the study population, there may be loss of information. All persons in this study were consecutively selected and had the same chance of inclusion in the interrater-analysis. There was fair resemblance between the study population and the study sample, helping to reduce sampling error.

Only few studies have examined agreement across several different spinal pathologies in the lumbar spine and with modest levels of reliability and high variability across pathologies (Fleiss κ and others). We achieved acceptable reliability for most degenerative pathologies ranging from: Gwet’s AC = 0.31–0.99.

In our study the raters were not selected randomly and only three specific raters were part of the analysis. In this study standard errors and confidence intervals of the study sample were based on fixed raters and consequently the inference generalizes and measures precision with respect to the universe of study persons only (with our inclusion criteria) and not the universe of raters, meaning its validity is limited to this specific group of raters that participated in the reliability experiment. This study indicated that even when raters have training materials or evaluation manuals available and use robust statistics, it is challenging to reach acceptable reliability for all degenerative pathologies.

We included no-LBP persons in our study sample for reliability, to resample the study population characteristics. This exposed us to the kappa paradoxes and made it difficult for us to compare our results directly to studies, where no-LBP persons were not included. But more importantly it challenged us to test the performance of several agreement coefficients, looking for more robust alternatives to Cohen’s and Conger’s extension of Cohen’s κ. Gwet’s AC_1_ and AC_2_ proved to be a good alternative. All no-LBP persons received supine MRI procedures in a new 0.5 T open MRI unit. This made it possible for raters to identify no-LBP persons, since the image quality was lower and certain sequences were used specifically for the 0.5 T unit. For the 1.5 T and 3 T systems, the sagittal T2 weighted sequence also included T2 fatsat (DIXON). It is possibly a source for bias of the raters to rate fewer findings in this group.

In this study the pair-wise comparison revealed that experienced chiropractors generally achieved the same levels of reliability as the medical radiologist. It is uncertain whether the lower average prevalence of positive findings, as reported by the radiologist, is due to underestimation or overestimation of the MRI-findings on part of the radiologist/chiropractors respectively. The chiropractors had similar reports of prevalence of positive finding, maybe due to the fact that they had collected the evidence and authored most of the evaluation guide. All raters had previous experience with reliability studies, but a high level of agreement among raters on negative findings has helped maintain acceptable reliability for most diagnostic findings in this study.

### Clinical and research implications

Reliability is an issue of concern, since it is of fundamental importance for the quality of health care, that raters or doctors can replicate and agree on their findings and conclusions [41]. In all health care disciplines doctors, researchers and others are working, at some level, with the concept of agreement and striving systematically to investigate healthcare procedures for their reliability and validity. Inter-rater reliability is an important parameter to measure and a concern as data-sets should reflect the study participants and not the raters involved in the study. For valid results it is important that raters are updated and trained in the use of standardized protocols prior to the evaluation of MRI findings. In this study two raters reported similar prevalence of positive findings, most likely due to more knowledge of the evaluation manual. More interdisciplinary reviews are needed to establish internationally recognized standards for degenerative spinal pathologies.

## Conclusion and recommendations

Inter-rater reliability for three rater-pairs was found overall acceptable for 6 of 8 degenerative MRI-findings of the lumbar spine. The two chiropractors in the study achieved similar levels of reliability as the medical radiologist for MRI interpretations of spinal degenerative pathologies. The classifications of findings are for most degenerative pathologies sufficiently comprehensible to be applied by health care professionals and can be used for both quality assurance and further research purposes. A few adjustments to the rating protocol will be required to bring all pathologies to an acceptable level of reliability.

## Supplementary information

**Additional file 1.** Prevalence of findings at disc level (ordinal)

**Additional file 2.** Prevalence of findings at disc level (nominal)

## Data Availability

The datasets used and/or analyzed during the current study are available from the corresponding author on reasonable request.

## Abbreviations

AC_1_: Agreement Coefficient (unweighted)
AC_2_: Agreement Coefficient (weighted)
CSF: Cerebrospinal Fluid
CT: Computerized Tomography
LBP: Low Back Pain
MRI: Magnetic Resonance Imaging
