# Supplementary material for: Degenerative findings in lumbar spine MRI: an inter-rater reliability study involving three raters
Source: Chiropr Man Therap. 2020 Feb 11;28:8. doi: 10.1186/s12998-020-0297-0 (PMC7011264; doi:10.1186/s12998-020-0297-0)
Supplement: Supplementary file 1 — Additional file 1. Prevalence of findings at disc level (ordinal) [file 12998_2020_297_MOESM1_ESM.pdf]

AgreeStat 2015.6.1 Recumbent MRI reliability study of 177 spinal levels (59 subjects)

MODULE: Chance-Corrected Agreement Coefficients (3 raters) (15. juni 2019)

Group: Spondylolisthesis **mr02**

DISTRIBUTION OF DISC LEVELS BY RATER AND SCORE/CATEGORY (59 subjects x 3 spinal levels x 3 directions(ant/ret/lat) = 531)

Category

Raters	0	1	2	3	Total
**Rater1**	527	4	0	0	531
**Rater2**	527	4	0	0	531
**Rater3**	524	7	0	0	531
**Average**	526	5	0	0	531,00

UNWEIGHTED ANALYSIS

Inter-Rater Reliability

METHOD	Coefficient	Inference/Subjects		
		**StdErr**	**95% C.I.**	**p-Value**
**Conger's Kappa**	0,32725	0,15131	0,03 to 0,624	3,100E-02
**Gwet's AC_1**	0,98737	0,00398	0,98 to 0,995	0,000E+00
**Percent Agreement**	0,98745	0,00394	0,98 to 0,995	0,000E+00

LANDIS-KOCH INTERPRETATION OF THE AGREEMENT COEFFICIENTS (Source of Variation: Subjects Only)

Benchmarking Unweighted Agreement Coefficients using Cumulative Membership Probabilities

Benchmark Scale	Interpretation	Conger' Kappa	Gwet AC_1	Percent Agreement
0,8 to 1	Almost Perfect	0,00089	1,00000	1,00000
0,6 to 0,8	Substantial	0,03572	1,00000	1,00000
0,4 to 0,6	Moderate	0,31533	1,00000	1,00000
0,2 to 0,4	Fair	0,79983	1,00000	1,00000
0 to 0,2	Slight	0,98472	1,00000	1,00000
Less than 0	Poor	1,00000	1,00000	1,00000

WEIGHTED ANALYSIS

Simple Ordinal Weights

	**0**	**1**	**2**	**3**
**0**	1	0,833333333	0,5	0
**1**	0,833333333	1	0,833333333	0,5
**2**	0,5	0,833333333	1	0,833333
**3**	0	0,5	0,833333333	1

Inter-Rater Reliability

WEIGHTED COEFFICIENTS

METHOD	**Coefficient**	**Inference/Subjects**		
		**StdErr**	**95% C.I.**	**p-Value**
**Conger's Kappa**	0,32725	0,15131	0,03 to 0,624	3,100E-02
**Gwet's AC_2**	0,99787	0,00068	0,997 to 0,999	0,000E+00
**Percent Agreement**	0,99791	0,00066	0,997 to 0,999	0,000E+00

LANDIS-KOCH INTERPRETATION OF THE AGREEMENT COEFFICIENTS (Source of Variation: Subjects Only)

Benchmarking Unweighted Agreement Coefficients using Cumulative Membership Probabilities

**Benchmark Scale**	**Interpretation**	**Conger' Kappa**	**Gwet AC_1**	**Percent Agreement**
0,8 to 1	Almost Perfect	0,00089	1,00000	1,00000
0,6 to 0,8	Substantial	0,03572	1,00000	1,00000
0,4 to 0,6	Moderate	0,31533	1,00000	1,00000
0,2 to 0,4	Fair	0,79983	1,00000	1,00000
0 to 0,2	Slight	0,98472	1,00000	1,00000
Less than 0	Poor	1,00000	1,00000	1,00000

DISTRIBUTION OF DISC LEVELS BY RATER AND SCORE/CATEGORY (59 subjects x 3 spinal levels = 177)

Raters	0	1	2	3	Total
**Rater1**	129	32	13	3	177
**Rater2**	105	53	19	0	177
**Rater3**	98	45	29	5	177
**Average**	110,7	43,3	20,3	2,7	177,00

(Columns 0–3: Category)

UNWEIGHTED ANALYSIS

Inter-Rater Reliability

METHOD	Coefficient	Inference/Subjects		
		StdErr	95% C.I.	p-Value
**Conger's Kappa**	0,52613	0,03416	0,459 to 0,594	0,000E+00
**Gwet's AC_1**	0,68820	0,03340	0,622 to 0,754	0,000E+00
**Percent Agreement**	0,74388	0,02530	0,694 to 0,794	0,000E+00

LANDIS-KOCH INTERPRETATION OF THE AGREEMENT COEFFICIENTS (Source of Variation: Subjects Only)
Benchmarking Unweighted Agreement Coefficients using Cumulative Membership Probabilities

Benchmark Scale	Interpretation	Conger' Kappa	Gwet AC_1	Percent Agreement
0,8 to 1	Almost Perfect	0,00000	0,00041	0,01328
0,6 to 0,8	Substantial	0,01530	0,99586	1,00000
0,4 to 0,6	Moderate	0,99989	1,00000	1,00000
0,2 to 0,4	Fair	1,00000	1,00000	1,00000
0 to 0,2	Slight	1,00000	1,00000	1,00000
Less than 0	Poor	1,00000	1,00000	1,00000

WEIGHTED ANALYSIS

Simple Ordinal Weights

	**0**	**1**	**2**	**3**
**0**	1	0,833333333	0,5	0
**1**	0,833333333	1	0,833333333	0,5
**2**	0,5	0,833333333	1	0,833333
**3**	0	0,5	0,833333333	1

WEIGHTED COEFFICIENTS

Inter-Rater Reliability

METHOD	**Coefficient**	**Inference/Subjects**		
		**StdErr**	**95% C.I.**	**p-Value**
**Conger's Kappa**	0,68039	0,03146	0,618 to 0,742	0,000E+00
**Gwet's AC_2**	0,90132	0,01488	0,872 to 0,931	0,000E+00
**Percent Agreement**	0,94978	0,00573	0,938 to 0,961	0,000E+00

LANDIS-KOCH INTERPRETATION OF THE AGREEMENT COEFFICIENTS (Source of Variation: Subjects Only)

Benchmarking Unweighted Agreement Coefficients using Cumulative Membership Probabilities

**Benchmark Scale**	**Interpretation**	**Conger' Kappa**	**Gwet AC_1**	**Percent Agreement**
0,8 to 1	Almost Perfect	0,00000	0,00041	0,01328
0,6 to 0,8	Substantial	0,01530	0,99586	1,00000
0,4 to 0,6	Moderate	0,99989	1,00000	1,00000
0,2 to 0,4	Fair	1,00000	1,00000	1,00000
0 to 0,2	Slight	1,00000	1,00000	1,00000
Less than 0	Poor	1,00000	1,00000	1,00000

Group: Nerve root compromise | mr09

DISTRIBUTION OF DISC LEVELS BY RATER AND SCORE/CATEGORY (59 subjects x 3 spinal levels = 177)

Category

Raters	0	1	2	3	Total
**Rater1**	158	13	3	3	177
**Rater2**	151	17	6	3	177
**Rater3**	135	28	5	9	177
**Average**	148	19,3	4,7	5	177,00

UNWEIGHTED ANALYSIS

Inter-Rater Reliability

METHOD	Coefficient	Inference/Subjects		
		**StdErr**	**95% C.I.**	**p-Value**
**Conger's Kappa**	0,36941	0,05326	0,264 to 0,475	7,399E-11
**Gwet's AC_1**	0,79797	0,02860	0,742 to 0,854	0,000E+00
**Percent Agreement**	0,81733	0,02403	0,77 to 0,865	0,000E+00

LANDIS-KOCH INTERPRETATION OF THE AGREEMENT COEFFICIENTS (Source of Variation: Subjects Only)

Benchmarking Unweighted Agreement Coefficients using Cumulative Membership Probabilities

Benchmark Scale	Interpretation	Conger' Kappa	Gwet AC_1	Percent Agreement
0,8 to 1	Almost Perfect	0,00000	0,47171	0,76458
0,6 to 0,8	Substantial	0,00001	1,00000	1,00000
0,4 to 0,6	Moderate	0,28288	1,00000	1,00000
0,2 to 0,4	Fair	0,99927	1,00000	1,00000
0 to 0,2	Slight	1,00000	1,00000	1,00000
Less than 0	Poor	1,00000	1,00000	1,00000

WEIGHTED ANALYSIS

Simple Ordinal Weights

	**0**	**1**	**2**	**3**
**0**	1	0,833333333	0,5	0
**1**	0,833333333	1	0,833333333	0,5
**2**	0,5	0,833333333	1	0,833333
**3**	0	0,5	0,833333333	1

WEIGHTED COEFFICIENTS

Inter-Rater Reliability

METHOD	**Coefficient**	**Inference/Subjects**		
		**StdErr**	**95% C.I.**	**p-Value**
**Conger's Kappa**	0,53952	0,06668	0,408 to 0,671	9,326E-14
**Gwet's AC_2**	0,93437	0,01361	0,908 to 0,961	0,000E+00
**Percent Agreement**	0,95166	0,00863	0,935 to 0,969	0,000E+00

LANDIS-KOCH INTERPRETATION OF THE AGREEMENT COEFFICIENTS (Source of Variation: Subjects Only)

Benchmarking Unweighted Agreement Coefficients using Cumulative Membership Probabilities

**Benchmark Scale**	**Interpretation**	**Conger' Kappa**	**Gwet AC_1**	**Percent Agreement**
0,8 to 1	Almost Perfect	0,00000	0,47171	0,76458
0,6 to 0,8	Substantial	0,00001	1,00000	1,00000
0,4 to 0,6	Moderate	0,28288	1,00000	1,00000
0,2 to 0,4	Fair	0,99927	1,00000	1,00000
0 to 0,2	Slight	1,00000	1,00000	1,00000
Less than 0	Poor	1,00000	1,00000	1,00000

DISTRIBUTION OF DISC LEVELS BY RATER AND SCORE/CATEGORY (59 subjects x 3 spinal levels x 5 sites (central, L+R foraminal, L+R recess) = 885)

	Category				
Raters	**0**	**1**	**2**	**3**	**Total**
**Rater1**	859	23	3	0	885
**Rater2**	806	68	11	0	885
**Rater3**	801	81	3	0	885
**Average**	822	57,3	5,7	0	885,00

UNWEIGHTED ANALYSIS

Inter-Rater Reliability

METHOD	**Coefficient**	**Inference/Subjects**		
		**StdErr**	**95% C.I.**	**p-Value**
**Conger's Kappa**	0,28277	0,03219	0,22 to 0,346	0,000E+00
**Gwet's AC_1**	0,89950	0,00872	0,882 to 0,917	0,000E+00
**Percent Agreement**	0,90395	0,00801	0,888 to 0,92	0,000E+00

LANDIS-KOCH INTERPRETATION OF THE AGREEMENT COEFFICIENTS (Source of Variation: Subjects Only)

Benchmarking Unweighted Agreement Coefficients using Cumulative Membership Probabilities

**Benchmark Scale**	**Interpretation**	**Conger' Kappa**	**Gwet AC_1**	**Percent Agreement**
0,8 to 1	Almost Perfect	0,00000	1,00000	1,00000
0,6 to 0,8	Substantial	0,00000	1,00000	1,00000
0,4 to 0,6	Moderate	0,00014	1,00000	1,00000
0,2 to 0,4	Fair	0,99493	1,00000	1,00000
0 to 0,2	Slight	1,00000	1,00000	1,00000
Less than 0	Poor	1,00000	1,00000	1,00000

WEIGHTED ANALYSIS

Simple Ordinal Weights

	**0**	**1**	**2**	**3**
**0**	1	0,833333333	0,5	0
**1**	0,833333333	1	0,833333333	0,5
**2**	0,5	0,833333333	1	0,833333
**3**	0	0,5	0,833333333	1

Inter-Rater Reliability

WEIGHTED COEFFICIENTS

METHOD	**Coefficient**	**Inference/Subjects**		
		**StdErr**	**95% C.I.**	**p-Value**
**Conger's Kappa**	0,32932	0,03874	0,253 to 0,405	0,000E+00
**Gwet's AC_2**	0,97991	0,00201	0,976 to 0,984	0,000E+00
**Percent Agreement**	0,98236	0,00159	0,979 to 0,985	0,000E+00

LANDIS-KOCH INTERPRETATION OF THE AGREEMENT COEFFICIENTS (Source of Variation: Subjects Only)

Benchmarking Unweighted Agreement Coefficients using Cumulative Membership Probabilities

**Benchmark Scale**	**Interpretation**	**Conger' Kappa**	**Gwet AC_1**	**Percent Agreement**
0,8 to 1	Almost Perfect	0,00000	1,00000	1,00000
0,6 to 0,8	Substantial	0,00000	1,00000	1,00000
0,4 to 0,6	Moderate	0,00014	1,00000	1,00000
0,2 to 0,4	Fair	0,99493	1,00000	1,00000
0 to 0,2	Slight	1,00000	1,00000	1,00000
Less than 0	Poor	1,00000	1,00000	1,00000

DISTRIBUTION OF DISC LEVELS BY RATER AND SCORE/CATEGORY (59 subjects x 3 spinal levels x 2 (L+R facet joint) = 354)

Category

Raters	0	1	2	3	Total
**Rater1**	233	107	0	14	354
**Rater2**	244	75	5	30	354
**Rater3**	223	92	11	28	354
**Average**	233,3	91,3	5,3	24	354,00

UNWEIGHTED ANALYSIS

Inter-Rater Reliability

METHOD	Coefficient	Inference/Subjects		
		StdErr	95% C.I.	p-Value
**Conger's Kappa**	0,20552	0,02790	0,151 to 0,26	1,251E-12
**Gwet's AC_1**	0,52879	0,02554	0,479 to 0,579	0,000E+00
**Percent Agreement**	0,60640	0,01933	0,568 to 0,644	0,000E+00

LANDIS-KOCH INTERPRETATION OF THE AGREEMENT COEFFICIENTS (Source of Variation: Subjects Only)

Benchmarking Unweighted Agreement Coefficients using Cumulative Membership Probabilities

Benchmark Scale	Interpretation	Conger' Kappa	Gwet AC_1	Percent Agreement
0,8 to 1	Almost Perfect	0,00000	0,00000	0,00000
0,6 to 0,8	Substantial	0,00000	0,00265	0,62974
0,4 to 0,6	Moderate	0,00000	1,00000	1,00000
0,2 to 0,4	Fair	0,57835	1,00000	1,00000
0 to 0,2	Slight	1,00000	1,00000	1,00000
Less than 0	Poor	1,00000	1,00000	1,00000

WEIGHTED ANALYSIS

Simple Ordinal Weights

	0	1	2	3
**0**	1	0,833333333	0,5	0
**1**	0,833333333	1	0,833333333	0,5
**2**	0,5	0,833333333	1	0,833333
**3**	0	0,5	0,833333333	1

Inter-Rater Reliability

WEIGHTED COEFFICIENTS

METHOD	**Coefficient**	**Inference/Subjects**		
		**StdErr**	**95% C.I.**	**p-Value**
**Conger's Kappa**	0,31521	0,04446	0,228 to 0,403	7,351E-12
**Gwet's AC_2**	0,78024	0,02126	0,738 to 0,822	0,000E+00
**Percent Agreement**	0,87979	0,00929	0,862 to 0,898	0,000E+00

LANDIS-KOCH INTERPRETATION OF THE AGREEMENT COEFFICIENTS (Source of Variation: Subjects Only)

Benchmarking Unweighted Agreement Coefficients using Cumulative Membership Probabilities

**Benchmark Scale**	**Interpretation**	**Conger' Kappa**	**Gwet AC_1**	**Percent Agreement**
0,8 to 1	Almost Perfect	0,00000	0,00000	0,00000
0,6 to 0,8	Substantial	0,00000	0,00265	0,62974
0,4 to 0,6	Moderate	0,00000	1,00000	1,00000
0,2 to 0,4	Fair	0,57835	1,00000	1,00000
0 to 0,2	Slight	1,00000	1,00000	1,00000
Less than 0	Poor	1,00000	1,00000	1,00000

Group: **Overall**

DISTRIBUTION OF DISC LEVELS BY RATER AND SCORE/CATEGORY

Raters	Category 0	1	2	3	Total
**Rater1**	1906	179	19	20	2124
**Rater2**	1833	217	41	33	2124
**Rater3**	1781	253	48	42	2124
**Average**	1840	216,3	36	31,7	2124,00

UNWEIGHTED ANALYSIS

Inter-Rater Reliability

METHOD	Coefficient	Inference/Subjects StdErr	95% C.I.	p-Value
**Conger's Kappa**	0,39215	0,01636	0,36 to 0,424	0,000E+00
**Gwet's AC_1**	0,84212	0,00720	0,828 to 0,856	0,000E+00
**Percent Agreement**	0,85468	0,00623	0,842 to 0,867	0,000E+00

LANDIS-KOCH INTERPRETATION OF THE AGREEMENT COEFFICIENTS (Source of Variation: Subjects Only)

Benchmarking Unweighted Agreement Coefficients using Cumulative Membership Probabilities

Benchmark Scale	Interpretation	Conger' Kappa	Gwet AC_1	Percent Agreement
0,8 to 1	Almost Perfect	0,00000	1,00000	1,00000
0,6 to 0,8	Substantial	0,00000	1,00000	1,00000
0,4 to 0,6	Moderate	0,31559	1,00000	1,00000
0,2 to 0,4	Fair	1,00000	1,00000	1,00000
0 to 0,2	Slight	1,00000	1,00000	1,00000
Less than 0	Poor	1,00000	1,00000	1,00000

WEIGHTED ANALYSIS

Simple Ordinal Weights

	0	1	2	3
**0**	1	0,833333333	0,5	0
**1**	0,833333333	1	0,833333333	0,5
**2**	0,5	0,833333333	1	0,833333
**3**	0	0,5	0,833333333	1

WEIGHTED COEFFICIENTS

Inter-Rater Reliability

METHOD	**Coefficient**	**Inference/Subjects**		
		**StdErr**	**95% C.I.**	**p-Value**
**Conger's Kappa**	0,50028	0,02321	0,455 to 0,546	0,000E+00
**Gwet's AC_2**	0,95376	0,00302	0,948 to 0,96	0,000E+00
**Percent Agreement**	0,96388	0,00209	0,96 to 0,968	0,000E+00

LANDIS-KOCH INTERPRETATION OF THE AGREEMENT COEFFICIENTS (Source of Variation: Subjects Only)

Benchmarking Unweighted Agreement Coefficients using Cumulative Membership Probabilities

**Benchmark Scale**	**Interpretation**	**Conger' Kappa**	**Gwet AC_1**	**Percent Agreement**
0,8 to 1	Almost Perfect	0,00000	1,00000	1,00000
0,6 to 0,8	Substantial	0,00000	1,00000	1,00000
0,4 to 0,6	Moderate	0,31559	1,00000	1,00000
0,2 to 0,4	Fair	1,00000	1,00000	1,00000
0 to 0,2	Slight	1,00000	1,00000	1,00000
Less than 0	Poor	1,00000	1,00000	1,00000
