# Supplementary material for: Degenerative findings in lumbar spine MRI: an inter-rater reliability study involving three raters
Source: Chiropr Man Therap. 2020 Feb 11;28:8. doi: 10.1186/s12998-020-0297-0 (PMC7011264; doi:10.1186/s12998-020-0297-0)
Supplement: Supplementary file 2 — Additional file 2. Prevalence of findings at disc level (nominal) [file 12998_2020_297_MOESM2_ESM.pdf]

AgreeStat 2015.6.1 Recumbent MRI reliability study of 177 spinallevels (59 subjects)

MODULE: Chance-Corrected Agreement Coefficients (3 raters) (Time: 12:54:02. Date: 16. juni 2019)

Group: Scoliosis | **mr05**

DISTRIBUTION OF DISC LEVELS BY RATER AND SCORE/CATEGORY

Category

Raters	0	1	2	3	4	Total
**Rater1**	172	5	0	0	0	177
**Rater2**	174	3	0	0	0	177
**Rater3**	172	5	0	0	0	177
**Average**	172,7	4,3	0	0	0	177,00

UNWEIGHTED ANALYSIS

Inter-Rater Reliability

		Inference/Subjects			Inference/Subjects & Raters		
METHOD	Coefficient	StdErr	95% C.I.	p-Value	StdErr	95% C.I.	p-Value
**Conger's Kappa**	0,60597	0,15446	0,301 to 0,911	1,251E-04	0,21439	0,184 to 1	4,975E-03
**Gwet's AC_1**	0,98094	0,00848	0,964 to 0,998	0,000E+00	0,01141	0,958 to 1	0,000E+00
**Percent Agreement**	0,98117	0,00833	0,965 to 0,998	0,000E+00	0,01123	0,959 to 1	0,000E+00

LANDIS-KOCH INTERPRETATION OF THE AGREEMENT COEFFICIENTS (Source of Variation: Subjects Only)

Benchmarking Unweighted Agreement Coefficients using Cumulative Membership Probabilities

Benchmark Scale	Interpretation	Conger' Kappa	Gwet AC_1	Percent Agreement
0,8 to 1	Almost Perfect	0,09968	1,00000	1,00000
0,6 to 0,8	Substantial	0,51279	1,00000	1,00000
0,4 to 0,6	Moderate	0,90832	1,00000	1,00000
0,2 to 0,4	Fair	0,99569	1,00000	1,00000
0 to 0,2	Slight	0,99996	1,00000	1,00000
Less than 0	Poor	1,00000	1,00000	1,00000

Group: Annular fissure | **mr06**

DISTRIBUTION OF DISC LEVELS BY RATER AND SCORE/CATEGORY

Category

Raters	0	1	2	3	4	Total
**Rater1**	163	14	0	0	0	177
**Rater2**	144	33	0	0	0	177
**Rater3**	148	29	0	0	0	177
**Average**	151,7	25,3	0	0	0	177,00

UNWEIGHTED ANALYSIS

Inter-Rater Reliability

METHOD	Coefficient	Inference/Subjects			Inference/Subjects & Raters		
		**StdErr**	**95% C.I.**	**p-Value**	**StdErr**	**95% C.I.**	**p-Value**
**Conger's Kappa**	0,52808	0,06514	0,4 to 0,657	8,482E-14	0,11342	0,305 to 0,751	4,574E-06
**Gwet's AC_1**	0,87561	0,02094	0,834 to 0,917	0,000E+00	0,02110	0,834 to 0,917	0,000E+00
**Percent Agreement**	0,88324	0,01910	0,846 to 0,921	0,000E+00	0,01947	0,845 to 0,922	0,000E+00

LANDIS-KOCH INTERPRETATION OF THE AGREEMENT COEFFICIENTS (Source of Variation: Subjects Only)

Benchmarking Unweighted Agreement Coefficients using Cumulative Membership Probabilities

Benchmark Scale	Interpretation	Conger' Kappa	Gwet AC_1	Percent Agreement
0,8 to 1	Almost Perfect	0,00001	0,99985	0,99999
0,6 to 0,8	Substantial	0,13479	1,00000	1,00000
0,4 to 0,6	Moderate	0,97536	1,00000	1,00000
0,2 to 0,4	Fair	1,00000	1,00000	1,00000
0 to 0,2	Slight	1,00000	1,00000	1,00000
Less than 0	Poor	1,00000	1,00000	1,00000

Group: Disc herniation type **mr08**

DISTRIBUTION OF DISC LEVELS BY RATER AND SCORE/CATEGORY

Category

Raters	0	1	2	3	4	Total
**Rater1**	144	22	10	0	1	177
**Rater2**	129	14	19	3	12	177
**Rater3**	101	41	34	1	0	177
**Average**	124,7	25,7	21	1,3	4,3	177,00

UNWEIGHTED ANALYSIS

Inter-Rater Reliability

METHOD	Coefficient	Inference/Subjects			Inference/Subjects & Raters		
		StdErr	95% C.I.	p-Value	StdErr	95% C.I.	p-Value
**Conger's Kappa**	0,34113	0,03663	0,269 to 0,413	0,000E+00	0,08027	0,183 to 0,499	2,743E-05
**Gwet's AC_1**	0,64381	0,03440	0,576 to 0,712	0,000E+00	0,08958	0,468 to 0,82	3,982E-12
**Percent Agreement**	0,68550	0,02810	0,63 to 0,741	0,000E+00	0,07349	0,541 to 0,83	0,000E+00

LANDIS-KOCH INTERPRETATION OF THE AGREEMENT COEFFICIENTS (Source of Variation: Subjects Only)

Benchmarking Unweighted Agreement Coefficients using Cumulative Membership Probabilities

Benchmark Scale	Interpretation	Conger' Kappa	Gwet AC_1	Percent Agreement
0,8 to 1	Almost Perfect	0,00000	0,00000	0,00002
0,6 to 0,8	Substantial	0,00000	0,89859	0,99883
0,4 to 0,6	Moderate	0,05400	1,00000	1,00000
0,2 to 0,4	Fair	0,99994	1,00000	1,00000
0 to 0,2	Slight	1,00000	1,00000	1,00000
Less than 0	Poor	1,00000	1,00000	1,00000

Group: **Overall**

DISTRIBUTION OF DISC LEVELS BY RATER AND SCORE/CATEGORY

Category

Raters	0	1	2	3	4	Total
**Rater1**	479	41	10	0	1	531
**Rater2**	447	50	19	3	12	531
**Rater3**	421	75	34	1	0	531
**Average**	449	55,3	21	1,3	4,3	531,00

UNWEIGHTED ANALYSIS

Inter-Rater Reliability

METHOD	Coefficient	Inference/Subjects			Inference/Subjects & Raters		
		StdErr	95% C.I.	p-Value	StdErr	95% C.I.	p-Value
**Conger's Kappa**	0,45264	0,03246	0,389 to 0,516	0,000E+00	0,08388	0,288 to 0,617	8,400E-08
**Gwet's AC_1**	0,83900	0,01439	0,811 to 0,867	0,000E+00	0,02843	0,783 to 0,895	0,000E+00
**Percent Agreement**	0,84997	0,01281	0,825 to 0,875	0,000E+00	0,02536	0,8 to 0,9	0,000E+00

LANDIS-KOCH INTERPRETATION OF THE AGREEMENT COEFFICIENTS (Source of Variation: Subjects Only)

Benchmarking Unweighted Agreement Coefficients using Cumulative Membership Probabilities

Benchmark Scale	Interpretation	Conger' Kappa	Gwet AC_1	Percent Agreement
0,8 to 1	Almost Perfect	0,00000	0,99664	0,99995
0,6 to 0,8	Substantial	0,00000	1,00000	1,00000
0,4 to 0,6	Moderate	0,94760	1,00000	1,00000
0,2 to 0,4	Fair	1,00000	1,00000	1,00000
0 to 0,2	Slight	1,00000	1,00000	1,00000
Less than 0	Poor	1,00000	1,00000	1,00000
